# Diagnosis and Treatment of Mitochondrial Myopathies

**DOI:** 10.1007/s13311-018-00674-4

**Published:** 2018-11-07

**Authors:** Syeda T. Ahmed, Lyndsey Craven, Oliver M. Russell, Doug M. Turnbull, Amy E. Vincent

**Affiliations:** 1 Wellcome Centre for Mitochondrial Research, Institute of Neuroscience, Newcastle University, Newcastle upon Tyne, UK; 2 MRC Centre for Ageing and Vitality, Newcastle University, Newcastle upon Tyne, UK

**Keywords:** Mitochondrial myopathy, diagnosis, treatment, mtDNA, muscle.

## Abstract

Mitochondrial myopathies are progressive muscle conditions caused primarily by the impairment of oxidative phosphorylation (OXPHOS) in the mitochondria. This causes a deficit in energy production in the form of adenosine triphosphate (ATP), particularly in skeletal muscle. The diagnosis of mitochondrial myopathy is reliant on the combination of numerous techniques including traditional histochemical, immunohistochemical, and biochemical testing combined with the fast-emerging molecular genetic techniques, namely next-generation sequencing (NGS). This has allowed for the diagnosis to become more effective in terms of determining causative or novel genes. However, there are currently no effective or disease-modifying treatments available for the vast majority of patients with mitochondrial myopathies. Existing therapeutic options focus on the symptomatic management of disease manifestations. An increasing number of clinical trials have investigated the therapeutic effects of various vitamins, cofactors, and small molecules, though these trials have failed to show definitive outcome measures for clinical practice thus far. In addition, new molecular strategies, specifically mtZFNs and mtTALENs, that cause beneficial heteroplasmic shifts in cell lines harboring varying pathogenic mtDNA mutations offer hope for the future. Moreover, recent developments in the reproductive options for patients with mitochondrial myopathies mean that for some families, the possibility of preventing transmission of the mutation to the next generation is now possible.

**Electronic supplementary material:**

The online version of this article (10.1007/s13311-018-00674-4) contains supplementary material, which is available to authorized users.

## Mitochondrial Disease

Mitochondrial myopathies are an important group of progressive muscle conditions, caused primarily by the impairment of oxidative phosphorylation (OXPHOS). OXPHOS is the biochemical process by which mitochondria produce energy in mammalian cells in the form of adenosine triphosphate (ATP). Myopathy is one of the most common manifestations of adult-onset mitochondrial disorders due to the high cellular energy demand of skeletal muscle. However, patients with mitochondrial myopathy often have dysfunction in multiple organ systems resulting in variability in clinical phenotype and prognosis [1].

Mitochondrial function is under the control of two genomes; the mitochondrial genome (mtDNA) and the nuclear genome (nDNA); as such, mitochondrial myopathy can be caused by pathogenic genetic variants in either of these genomes. This dual genetic control also means that mitochondrial disease is transmitted with the following inheritance patterns: maternal (mtDNA), X-linked, autosomal recessive, autosomal dominant. In addition, some relatively common mitochondrial myopathies occur *de novo.*

The mitochondrial genome is a small, circular DNA molecule that encodes 13 proteins of the OXPHOS machinery, 22 mitochondrial tRNA (mt-tRNA), and 2 mitochondrial rRNAs (mt-rRNA). Primary mutations of the mtDNA include point mutations affecting protein coding regions of the genome and mt-tRNA genes which alter mitochondrial protein synthesis. They also include single, large-scale mtDNA deletions which can either be inherited or arise sporadically during embryogenesis [2]. The pathogenicity of mtDNA mutations is further complicated by heteroplasmy (the state in which there is co-existence of both mutant and wild-type mtDNA in a given cell) and the threshold effect (when the proportion of mutant mtDNA exceeds a given limit and causes a biochemical defect in a cell) [3].

The nDNA encodes 1171 known and 442 predicted mitochondrial proteins (MitoMiner v.Q2 2018 [4]), including OXPHOS subunits, assembly factors for OXPHOS complexes and proteins required for mtDNA maintenance. Mutations in the genes encoding the mtDNA maintenance machinery can lead to either mtDNA depletion or multiple mtDNA deletions*.*

### Clinical Features of Mitochondrial Myopathies

**Fig. 1:**
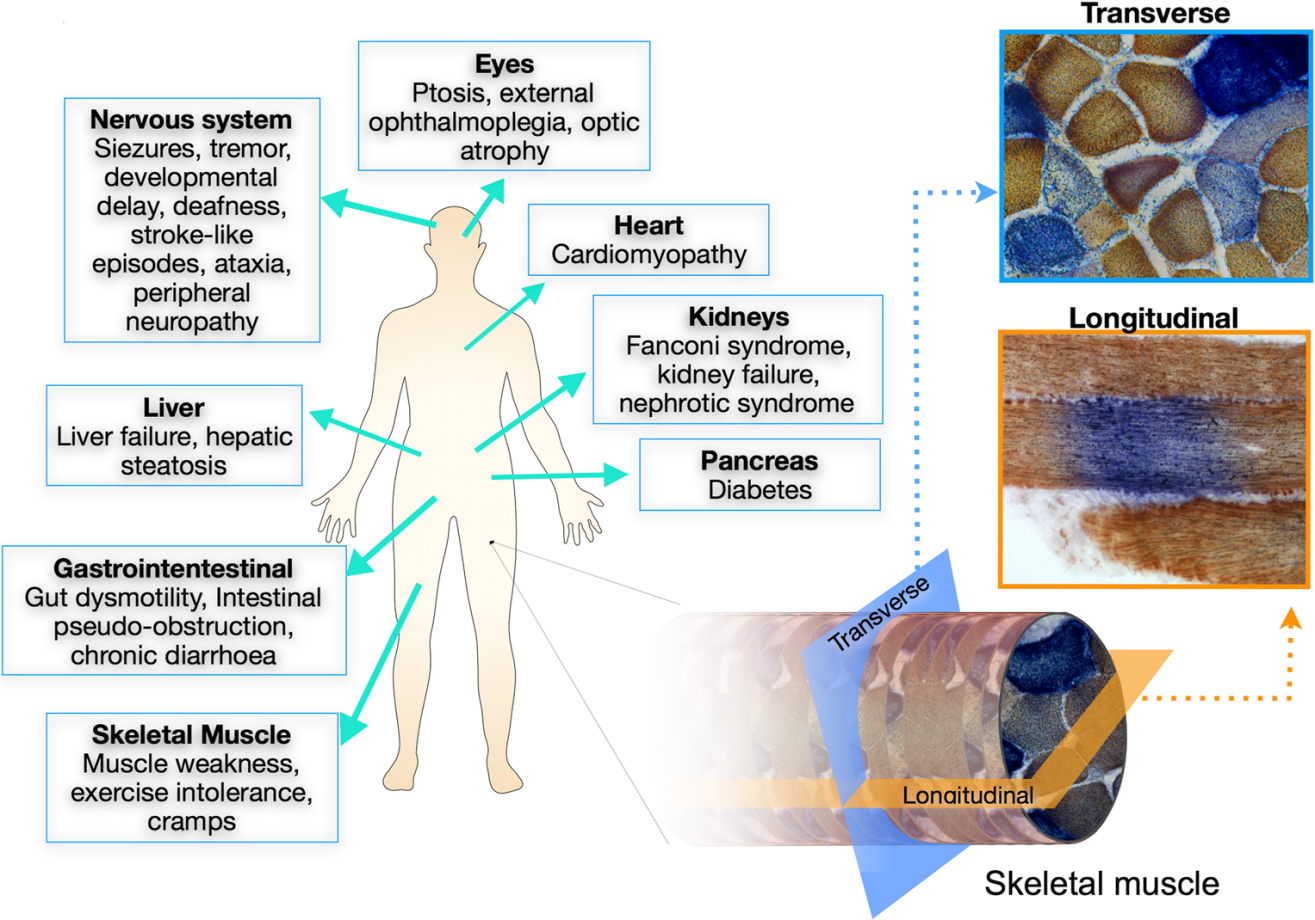
Mitochondrial disease symptoms and skeletal muscle biopsies. Mitochondrial diseases are multisystemic disorders that present with a wide variety of neurological, muscular, hepatic, and gastrointestinal symptoms, among others (left). Myopathy is associated with mitochondrial disease and often leads to exercise intolerance, cramps, and fatigue. Skeletal muscle biopsies are commonly taken for diagnostic purposes. When reacted for COX/SDH histochemistry, a mosaic pattern of affected (COX-deficient) and unaffected (COX-positive) muscle fibers can be seen in transverse sections (light blue). When muscle biopsies are examined longitudinally, COX-deficiency can be seen restricted to small segments along the length of the fiber surrounded by COX-positive regions (orange)

Patients with mitochondrial myopathies have diverse clinical phenotypes (Fig. 1), some features may be similar to other myopathies and others are more specific for patients with mitochondrial disease. In virtually all patients with mitochondrial myopathy, there is potential involvement of other systems which may well be the prominent and life-threatening feature—for example, cardiomyopathy, epilepsy, or stroke-like episodes (Fig. 1) [1]. Indeed the most frequent presentation of mitochondrial myopathy is in combination with other symptoms which is often the clue to likely mitochondrial involvement. While beyond the scope of this review to cover these features, it is essential that they are considered when evaluating all patients.

*Chronic progressive external ophthalmoplegia (CPEO)* is a common presentation for patients with mitochondrial disease [5] and usually involves progressive ptosis and a slow progressive ophthalmoplegia with or without double vision. CPEO can occur either as an isolated symptom or as part of a multisystem disease such as Kearns–Sayre syndrome and is commonly associated with proximal myopathy.

*Proximal myopathy* is the most common form of myopathy in mitochondrial disease patients. The degree of weakness is variable and commonly fatigable. In some patients, this weakness is progressive and can affect diaphragm and/or respiratory muscles, eventually requiring ventilator support.

*Exercise-induced muscle pain* is a common feature, limiting exercise tolerance. Rarely, this can be associated with rhabdomyolysis, which should be included in the differential diagnosis of mitochondrial myopathies.

*Fatigue* is the most commonly patient-reported symptom [6]. Interestingly, in a survey of patients, Gorman et al. found fatigue to be associated with exercise intolerance, difficulty swallowing, cutting food and dressing, hygiene, gait, and psychiatric symptoms but not with myopathy [6].

### Diagnosis of Mitochondrial Myopathies

The diagnosis of mitochondrial myopathies involves a multidisciplinary approach. History and physical examination are crucial for recognizing that mitochondrial myopathy is a potential diagnosis but also to suggest the most appropriate diagnostic studies. The diagnostic investigations include histological and immunohistochemical studies, enzymatic analysis of the OXPHOS complexes, and the genetic analysis of the mtDNA. Additionally, if a nuclear genetic diagnosis is suspected, a targeted nDNA sequencing approach may be used. If no pathogenic mutation is identified, whole genome and whole exome screens are now commonly used to search for potential genetic diagnoses, with new disease genes constantly being identified. By integrating the information from these diagnostic tests, it allows for a diagnosis in the majority of patients.

#### Muscle Biopsy

Skeletal muscle is commonly affected in mitochondrial disease and is the most frequently biopsied tissue, although the increasing use of genetic tests is likely to reduce the need for muscle biopsies in the future [7]. Patients with mitochondrial myopathy may show histochemical alterations in their skeletal muscle which indicate mitochondrial dysfunction, although in some patients, the muscle biopsy can appear normal. These mitochondrial abnormalities can be identified using several routine histological and immunological studies.

One example detected in some patients is the staining of skeletal muscle cryosections with the modified Gomori Trichrome, highlighting the presence of ragged-red fibers (RRF). These fibers can also be detected using succinate dehydrogenase (SDH, complex II) histochemistry, which detects the mitochondrial aggregates in the subsarcolemmal region of the fiber due to mitochondrial proliferation that occurs as a result of mitochondrial OXPHOS dysfunction [8].

Another important diagnostic feature of mitochondrial myopathy is the presence of cytochrome c oxidase (COX, complex IV)-negative fibers as detected by the sequential COX/SDH histochemistry. A mosaic pattern is commonly observed with COX-negative fibers appearing blue, among the normal COX-positive fibers which appear brown (Fig. 1). Furthermore, COX deficiency is segmental along the length of the muscle fiber (Fig. 1). The mosaic pattern is due to different levels of mutational heteroplasmy with high mutation load leading to respiratory chain deficiency [9, 10]. Mutation load is known to increase in cells throughout life, a process termed clonal expansion, which means that healthy aged individuals accumulate a low frequency of COX-negative fibers [9, 11]. However, a suggestive diagnosis of mitochondrial myopathy is only made when individuals harbor COX-negative fibers at a frequency of > 5%.

While COX/SDH fails to provide information on complex I deficiency, a more recently established quadruple immunofluorescent assay [13], has been shown to effectively identify isolated complex I downregulation in skeletal muscle biopsies of patients for diagnostic purposes. These patients had a confirmed genetic diagnosis in a nDNA-encoded subunits of complex I or assembly factors, or mutations in the mtDNA complex I subunits, with some patients having mitochondrial myopathy [12, 13].

Other pathological features which may be seen in skeletal muscle are nonspecific. These include neurogenic atrophy, internal nuclei, abnormal variation in fiber size, and accumulations of glycogen or lipid.

#### Biochemical Studies

Spectrophotometric evaluation of individual respiratory chain complex activities is an important approach to the biochemical investigation and diagnosis of mitochondrial myopathies. It can be performed in either fresh or frozen muscle homogenate; however, the latter is more common in diagnostic centers due to cross-continental or national referral of patients. Each complex can be analyzed in isolation following the oxidation/reduction of specific substrates or substrate analogs. The spectrophotometric enzyme assays are as follows: NADH:ubiquinone oxidoreductase for complex I, succinate:cytochrome *c* oxidase oxidoreductase for complex II, ubiquinol cytochrome c oxidoreductase for complex III, cytochrome *c* oxidase for complex IV. The measurement of complex V (oligomycin-sensitive ATP synthase) is more challenging and requires the use of fresh material and is often measured in cultured skin fibroblasts. Bernier et al. [14] recommended 20–30% of normal complex activities as a criterion for the diagnosis of mitochondrial myopathies. However, a normal respiratory chain enzyme activity does not exclude the diagnosis of mitochondrial myopathies as a small percentage of respiratory chain deficiency cells may not be detectable by enzyme measurements on tissue homogenates.

Another biochemical study which proves to be a helpful step in the diagnosis of mitochondrial myopathy is the blue native acrylamide page (BN-PAGE) assay, which is used to assess the relative abundance of fully assembled respiratory chain enzyme complexes. Similar to the spectrophotometric assay, deficiency of the complexes can be seen as an isolated single complex or a combined deficiency.

#### Molecular Genetics Studies

While histochemical and biochemical studies pave the way for appropriate molecular genetic testing, the diagnostic procedure in mitochondrial diseases has shifted towards the “genetic first approach” because of the advances made with next-generation sequencing (NGS) [15]. The application of NGS techniques including targeted multi-gene panels of candidate genes, unbiased exome sequencing and whole genome sequencing, has revolutionized the diagnostic approach, replacing the sequential method of sequencing candidate genes through Sanger sequencing as the first diagnostic approach. The high-throughput analysis of many genes has led to an increase in the pace of diagnosis and reducing costs. Moreover, unlike Sanger sequencing, NGS can be used for determination of mtDNA heteroplasmy and deletions, although real-time PCR, pyrosequencing, and long-range PCR are still commonly used. The identification of a causative molecular defect facilitates a diagnosis of mitochondrial myopathy in the patient and family members, permitting disease management, genetic counselling, and a variety of reproductive options.

#### Molecular Studies to Identify Causative Genes

**Fig. 2:**
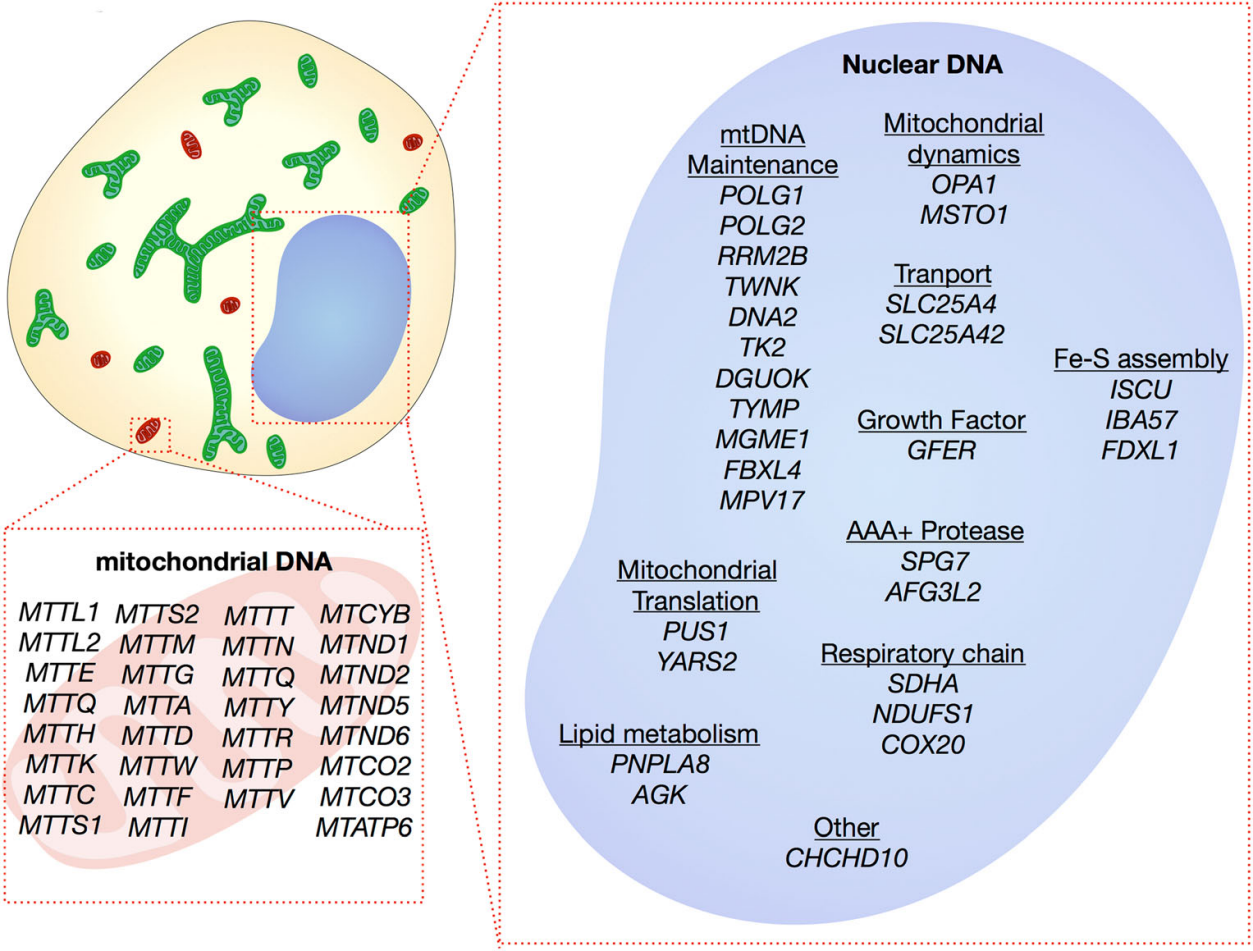
Molecular genetics of mitochondrial myopathy. A search of OMIM, ClinVar, and Mitomap from genes causing mitochondrial myopathy yielded 60 genes. Of these, 31 are mitochondrial genes, including 8 protein-encoding and 23 tRNA mutations. The remaining 29 genes are encoded by the nucleus and have a variety of functions, including subunits and assembly factors of the respiratory chain, mtDNA replication and maintenance, mitochondrial shape, mitochondrial translation, transport, proteases, lipid metabolism, and iron sulphur (Fe-S) cluster formation. Due to the speed at which needed genes are identified, this is likely to be an underestimate of causative genes.

Mutations causing mitochondrial myopathy can be found in either the mtDNA or nDNA (Fig. 2). For mitochondrial disease in general, pathogenic mutations have been reported in all 37 mtDNA genes and more than 254 nDNA-encoded genes [16]. A search of OMIM, ClinVar, and MitoMap yielded 31 mtDNA genes and 29 nDNA genes that are associated with mitochondrial myopathy at time of writing. However, with new disease genes being identified at a rapid rate, this is likely to be an underestimate and the muscle is often involved in many of the more systemic mitochondrial diseases.

When clinical history shows evidence of a maternally inherited disorder, or a typical mitochondrial myopathy clinical phenotype, the entire mitochondrial genome is typically analyzed. NGS allows for deep coverage across mtDNA and thus detection of low levels of heteroplasmy, point mutations, and breakpoints of single, large-scale mtDNA deletions. Analysis can be undertaken from mtDNA extracted from blood, skin biopsy, or muscle. However, possible mitotic segregation or indeed a loss of mtDNA mutation from mitotic tissues [17] means that if an mtDNA mutation is strongly suspected, then analysis of muscle mtDNA is recommended.

When the compilation of various information from the clinical history and histochemical and biochemical tests indicate that the pathogenic mutation is located in the nDNA, whole exome sequencing (WES), whole genome sequencing (WGS) or a targeted multigene panel of candidate genes can be employed to identify the causative gene using DNA extracted from blood. Due to the large number of nuclear-encoded mitochondrial genes the benefits of NGS are obvious [18, 19].

In the case of novel mutations that have not been previously reported, functional tests must be undertaken to confirm the pathogenicity of the defect. These typically include looking at pathogenicity predictions based on protein structure and “rescue” experiments where patient cells are transfected with a wild-type copy of the suspected gene and this is shown to rescue the disease phenotype.

#### Additional Molecular Studies of mtDNA

For some patients with mitochondrial myopathy, it is helpful to determine mtDNA copy number in muscle tissue using real-time PCR. If the mtDNA content is depleted, it indicates that the defect has occurred in a nuclear gene responsible for mtDNA replication and/or maintenance of the deoxynucleotide pools and thus may be helpful for targeted nuclear genetic testing if not already completed [20].

Real-time PCR and long-range PCR may be used if a single, large-scale mtDNA deletion or multiple mtDNA deletions are suspected. Single, large-scale mtDNA deletions are most reliably detected in muscle and through determining the size of deletion and heteroplasmy provides important information as regards to disease severity and progression [21]. In comparison, the presence of multiple mtDNA deletions indicates a genetic defect in a nuclear gene involved in mtDNA maintenance and provides guidance for nDNA sequencing.

#### Other Investigations into Mitochondrial Myopathies

##### Lactate–Pyruvate

Lactate is a product of anaerobic glucose metabolism which accumulates when the metabolism is impaired, causing a shift in the oxidized-to-reduced NAD+/NADH ratio within the mitochondria—indicated by the blood lactate–pyruvate ratio. The determination of lactate concentrations at rest or following exercise has become a diagnostic tool for mitochondrial myopathy [22, 23]. However, many patients do present with consistently normal lactate–pyruvate ratio especially in adults.

##### Fibroblast Growth Factor 21

Fibroblast growth factor 21 (FGF-21), which has a regulatory role in lipid metabolism, can also be used as a blood serum biomarker for mitochondrial diseases. FGF-21 levels are seen to be raised in patients who have muscle involvement, and thus, in cases with suspected mitochondrial myopathies, the noninvasive measurement of the growth factor acts as a helpful and sensitive first-line investigation in the diagnostic process [24, 25]. Furthermore, FGF-21 specificity for mitochondrial disease has been determined to be above 90% [26].

##### Growth Differentiation Factor 15

More recently, the growth differentiation factor 15 (GDF-15), which is a member of the transforming growth factor β superfamily, is shown to be significantly higher in the serum of patients with mitochondrial disease [27, 28]. The serum marker has been seen to be associated with disease severity and so is deemed the most useful biomarker for mitochondrial diseases [27].

##### Exercise Test

The use of exercise testing, usually by bicycle or treadmill, is used for research and as a clinical diagnostic test for mitochondrial myopathy. Exercise intolerance during clinical observation can be demonstrated through taking venous blood sampling both during and after exercise. The test highlights a potential increase in concentration of muscle metabolites (lactate and pyruvate) in venous blood supply (measured as systemic arteriovenous oxygen (a-vO2)) during post-exercise recovery and a slow clearance of the accumulated plasma lactate. The aerobic forearm test is also used as a screening tool for mitochondrial myopathy, coupled with the venous oxygen saturation measurements. In a patient with mitochondrial myopathy, the combined test will reveal a decrease in oxygen desaturation in the venous blood as mitochondrial dysfunction in skeletal muscle results in its inability to extract oxygen from blood [29].

### Treatment for Mitochondrial Myopathies

There are currently no effective or disease-modifying treatments available for the majority of patients with mitochondrial myopathies. Instead, existing therapeutic options focus on the symptomatic management of disease manifestations, helping to improve the patients’ quality of life. Disease management is tailored to the individual patient according to their specific needs and requires the input of many different healthcare professionals such as neurologists, endocrinologists, cardiologists, dieticians, speech and language therapists, and physiotherapists. Treatment guidelines are in place to help provide for the management of patients in a clinical setting (for further information, see the guidelines available at: http://www.newcastle-mitochondria.com/clinical-professional-home-page/clinical-publications/clinical-guidelines/).

**Fig. 3:**
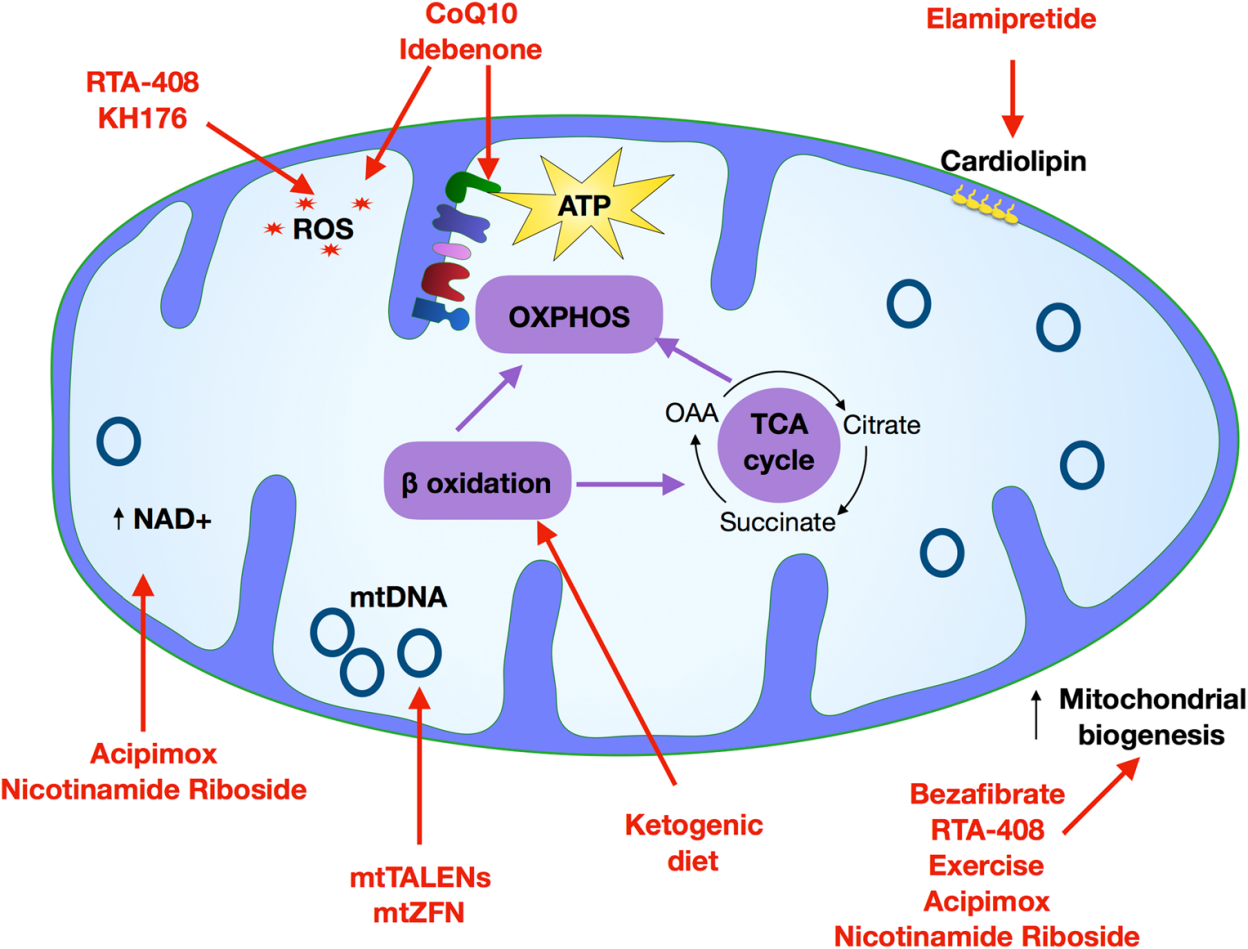
Routes to treatment. Schematic depicts the mitochondrial targets of treatments currently being tested for use in treating mitochondrial myopathy. A wide range of approaches are currently in use including the targeting of mitochondrial biogenesis (bezafibrate, RTA-408, exercise, acipimox and nicotinamide riboside), oxidative phosphorylation (CoQ10, idebenone, riboflavin and thiamine), ROS (RTA-498, KH176, CoQ10, idebenone), increasing NAD+ (acipimox and nicotinamide riboside), mtDNA gene editing (mitochondrial TALENS and zinc finger nucleases), lipid utilization (ketogenic diet), and cardiolipin stabilization (elamipretide)

An increasing number of clinical trials, usually designed to be double blinded and placebo controlled, have investigated the therapeutic effects of various vitamins, cofactors, and nutritional supplements, though often these trials have failed to show definitive beneficial primary and secondary outcomes (referred to in this review). Moreover, new molecular and cellular strategies are being proposed that act on a molecular or cellular level, for example restriction endonucleases technologies. Treatment options are described below and both previous and ongoing clinical trials are summarized in Table 1, and their molecular taregts detailed in Fig. 3.

**Table 1:** Treatment options for mitochondrial myopathies

Treatment	Target of intervention	Clinical Trial identifier	Clinical phase	Outcome
CoQ10	Increase respiratory chain flux, serve as antioxidant	NCT00432744	Phase 3	Completed—no data available
Idebenone	Serve as antioxidant	NCT00887562	Phase 2	Primary endpoint did not reach a significant endpoint at the completion of study
		NCT00747487	Phase 2	Improvement in secondary outcome measures in patients with LHON patients with discordant visual acuities
Nicotinamide riboside (NR)		NCT03432871	N/A	Recruiting—no data available.
Acipimox		NCT00943059		2-week treatment, dose of 250 mg three times a day, showed increase in skeletal muscle mitochondrial oxidative capacity. An improvement in the ATP production
Bezafibrate	Mitochondrial biogenesis	NCT02398201	Phase 2	Change in respiratory enzyme activity
RTA-408	NRF2 activator, NFĸB inhibitor, antioxidant	NCT02255422	Phase 2	Completed—no data available
Exercise		NCT00457314	Phase 2	N/A (status of study unknown)—no data available
MTP-131	Cardiolipin stabilization	NCT02367014	Phase 1/2	Improved exercise intolerance and walking distance in 6 min walk test
		NCT02805790	Phase 2	Ongoing
		NCT02976038	Phase 2	Ongoing
KH176	Antioxidant	NCT02909400	Phase 2	Ongoing

#### Ubiquinone and Ubiquinone Analog

CoQ10, an electron transport chain component involved in shuttling electrons from complex I and complex II to complex III via the quinone pool, has been shown to be an effective treatment option in patients who harbor a rare congenital CoQ10 deficiency [30]. The compound has been evaluated in a phase 2 clinical trial which included adult patients with either myoclonic epilepsy, lactic acidosis, and stroke-like episodes (MELAS), Leber’s hereditary optic neuropathy (LHON), or CPEO. The study reported minor improvements in the aerobic capacity and post-exercise lactate levels, but there were no improvement in clinical measures such as strength or resting lactate [31]. A phase 3 trial has also been completed which aimed to show that oral CoQ10 was a safe effective treatment for children with inherited mitochondrial disease caused by defects in specific respiratory chain complexes or mtDNA mutations (Clinical Trial identifier: NCT00432744). No results have been reported as of yet [32].

Idebenone, a synthetic quinone analog of CoQ10, may have the potential to restore cellular ATP generation [33]. It also acts as an antioxidant by protecting the lipid membrane and mitochondria from oxidative damage. Idebenone was used in a randomized controlled trial (Clinical Trial identifier: NCT00747487) which showed an improvement in secondary outcome measures in LHON patients with discordant visual acuities [34]. Following this, LHON became the first mitochondrial disease for which a treatment has been approved by the European Medicine Agency. Idebenone was also investigated in a phase 2 clinical trial (Clinical Trial identifier: NCT00887562) at two different doses of either 900 mg or 2400 mg in MELAS patients [35, 36]. On the completion of the trial, the results reached no statistical significance.

#### Targeting Mitochondrial Biogenesis

Nicotinamide riboside (NR), a form of vitamin B3 and a natural precursor of NAD+, has been shown to be a promising treatment strategy for mitochondrial myopathy. NR increases the levels of NAD+ which has been shown to induce mitochondrial biogenesis, thus increasing oxidative ATP production capacity [37, 38]. A study by Khan et al. [39], investigated the effect of NR using Deletor mice which have a mutation in the mtDNA Twinkle (TWNK) helicase [40]. Multiple mtDNA deletions accumulate in the skeletal muscle from these mice causing COX-negative fibers and eventually myopathy after the age of 12 months. The study administered NR at a dose of 400 mg/kg/day to all control, presymptomatic, and postsymptomatic mice for a period of 4 months, a regime which had already proven to increase levels of NAD+ in skeletal muscle of wild-type mice [37]. The findings showed that NR prevented both the development and progression of mitochondrial myopathy. The treatment also resulted in a significant induction of mitochondrial biogenesis and oxidative metabolism, similar to the findings of previous studies [37, 38]. The increased biogenesis resulted in a delayed development of morphological hallmarks, ultrastructural abnormalities, and the prevention of mtDNA deletion accumulation in skeletal muscle. A more recent clinical trial is underway investigating NR supplementation in patients with mitochondrial disease to induce mitochondrial biogenesis (Clinical Trial identifier: NCT03432871) but results are yet to be confirmed at the completion of the trial. Further research is also underway to improve the uptake of NR into patient blood stream by providing patients with a potential modified release oral supplement or intravenous version of NR.

A NAD+ precursor compound named acipimox has also been tested in an interventional clinical trial study which recruited patients with type 2 diabetes mellitus (Clinical Trial identifier: NCT00943059). Findings from the trial showed that a 2-week treatment with 250 mg of acipimox three times daily resulted in an increase in skeletal muscle mitochondrial oxidative capacity and an improvement in the ATP production. Though the researchers believe further insight need to be given into the safety and efficacy of the compound as a potential treatment option [41]. An upcoming trial for acipimox is soon to start in Newcastle, UK.

Bezafibrate is a pan-PPAR agonist that stimulates PGC1α, a coactivator responsible for the induction of mitochondrial biogenesis through its interaction with a number of transcription factors. Preclinical studies undertaken on various mouse models have reported varied findings as to whether bezafibrate could be a potential therapeutic strategy for mitochondrial myopathies. One study on Deletor mice showed that bezafibrate treatment, starting at the time of disease manifestation (12 months of age), resulted in a milder progression of myopathy and reduced COX-negative fibers and mtDNA deletions in skeletal muscle [42]. In contrast, studies using the Surf1-KO mice (a model of early-onset partial COX-deficiency) and the COX15^−/−^ mice showed no change in COX-activity, percentage COX-deficient fibers, and, importantly, no induction of mitochondrial biogenesis. Rather, the studies showed a suppression with no increase in the PPARs or PGC1α expression following treatment [42, 43]. However, both studies did highlight the adverse effects of the drug on mice, mainly severe lipid metabolism side effects and hepatomegaly. Despite these findings, two clinical trials are currently ongoing for bezafibrate treatment. The first is a randomized placebo-controlled trial aiming to assess the safety and efficacy of bezafibrate in mitochondrial myopathy patients (EudraCT number: 2012-002692-34). The trial is administrating bezafibrate orally at a dose of 200 mg in patients aged 2 to 50 years. The second trial, which is a phase 2, open labelled feasibility study is aiming to provide proof of the effects of bezafibrate on six patients with the m.3243A > G mutation (Clinical Trial number: NCT02398201). The results of these clinical trials are not yet published.

RTA-408 is a synthetic triterpenoid compound and a potent activator of nuclear factor erythroid 2-related factor 2 (Nrf2), working to increase the cellular antioxidant response [44]. Nrf2 is a transcriptional target of PGC1α and Nrf2 which promotes mitochondrial biogenesis [45]. Therefore, RTA-408 has the potential to improve muscle function, oxidative phosphorylation, antioxidant capacity, and mitochondrial efficiency in patients with mitochondrial myopathy. The drug form of RTA-408 is called omaveloxolone which has been investigated in a recently completed phase 2, clinical trial (in a total of 53 patients with mitochondrial myopathy (Clinical Trial number: NCT02255422—known as the MOTOR trial); however, the results are not yet published.

Ketogenic diets, which consist of a low carbohydrate and high lipid content which helps the lipid utilization by the mitochondria, have been proposed as a possible treatment for mitochondrial myopathies. The diet was found to simulate mitochondrial oxidative metabolism in Deletor mice, reducing the amount of COX-negative fibers, preventing mitochondrial ultrastructural abnormalities in the skeletal muscle and inducing mitochondrial biogenesis [46]. Another preclinical trial found that a ketogenic culture medium killed cybrid cell lines carrying mtDNA mutation derived from a heteroplasmic patient with a single, large-scale mtDNA deletion. A reduction in the mtDNA deletion load was detected in the heteroplasmic cells line indicative of a heteroplasmic shift [47]. Furthermore, Ahola et al. [48] tested a ketogenic diet called the modified Atkins Diet (mAD) in five patients with mitochondrial myopathy with either single or multiple mtDNA deletions. Results of the pilot study showed no induction of mitochondrial biogenesis. A 2-year follow-up of the patients revealed an improvement in muscle strength, showing a potential activation of muscle regeneration. Despite the positive outcomes, further work is warranted to determine whether ketogenic diets can have a therapeutic effect on patients with mitochondrial myopathy.

#### Targeting the Regulation of Lipid Dynamics

Elamipretide (previously Bendavia), is a member of the Szeto-schiller (SS) family. The drug targets the mitochondrial intermembrane lipid cardiolipin [49], working to stabilize the lipid structure. Promising findings from preclinical trials have shown that the compound led to an increase in the OXPHOS efficiency through a reduction of ROS generation and so ultimately an increase in ATP production [50, 51]. The drug has been assessed in mitochondrial myopathy patients (Clinical Trial identifier: NCT02367014, known as MMPOWER) and recently published data from the trial showed that patients had improved exercise intolerance and walking distance after the administration of the drug at the highest dose [52]. These findings have led to an ongoing extension trial (Clinical Trial identifier: NCT02976038, MMPOWER2).

#### Nutritional Supplementations

Another small molecule being investigated for treatment of mitochondrial myopathy is KH176, a vitamin E derivative which acts a potent ROS scavenger. An initial dose–dependent study conducted by Koene et al. [53] showed that the molecule was tolerated at a dose of 800 mg in male participants, causing clinically relevant changes to cardiac electrophysiology tests. A phase 2 clinical trial is currently being undertaken to investigate this further (Clinical trial identifier: NCT02909400).

#### Exercise

It is well documented that exercise programs (either aerobic, endurance, or resistance) can provide a safe therapeutic option to patients with mitochondrial myopathy, benefiting the biochemical (increasing phosphocreatine synthesis and mitochondrial enzymes) and clinical (work capacity, fatigue, quality of life, and strength) end points [54–61].Exercise has been shown to promote mitochondrial biogenesis through activation of PGC1α, AMPK, P38Ɣ, MAPK, and RCG-1β. However, it is not yet clear if exercise is having an effect on the underlying pathogenesis of mitochondrial myopathy or reversing the deconditioning of the muscle. A phase 2 exercise trial consisting of 50 patients with mitochondrial myopathy is currently ongoing (Clinical Trial identifier: NCT00457314) and results are yet to be confirmed. It should, however, be remembered that the benefits of exercise will be limited to those patients that are physically able; thus, development of exercise-mimetic drugs is desirable.

#### Other Potential Treatment Options for Mitochondrial Myopathies

The development of alternative strategies that act on a molecular level to manipulate the mitochondrial genome is more recently being proposed as therapeutic options for mitochondrial diseases. This manipulation of mtDNA is achieved with the use of restriction endonucleases, engineered specifically for the mitochondria. The approaches are designed to recognize and bind to specific mtDNA sequences, induce a double-strand break, and initiate targeted degradation of the mutant mtDNA in a heteroplasmic population. One such approach involves the use of mitochondrially targeted zinc finger nucleases (mtZFN), a heterodimer nuclease consisting of a DNA binding site (tandem repeat of zinc fingers binding three bases each), and a DNA cleavage domain (a Fok1 endonuclease). A further development to mtZFN is mitochondrially targeted transcription activator like effectors (TALE) fused with a Fok1 nuclease (together abbreviated to mtTALENs). Studies have shown the ability of both mtZFNs and mtTALENs to selectively eliminate mutant mtDNA in cell lines harboring a number of pathogenic mutations, including the mtDNA point mutations m.8993T > G (associated with neuropathy, ataxia, and retinitis pigmentosa (NARP)), m.8344A > G (associated with myoclonic epilepsy with ragged-red fibers (MERRF)), m.13513A > G MT-ND5 mutation (associated with MELAS and Leigh syndrome (LS)) and also the “common deletion” m.8483-1345del4977 (associated with CPEO and Kearns–Sayre syndrome (KSS)). These studies showed that through the targeted elimination of mutated mtDNA, a beneficial shift in heteroplasmy and improvement in the biochemical deficit can be achieved [62–67]. However, despite the encouraging findings, these techniques are limited by their lack of efficacy in the delivery mechanism to affected tissues. Once this issue has been resolved, gene-editing techniques could offer a potential treatment option for patients with primary mutations in the mitochondrial genome.

Deoxypyramidine bypass therapy has been trialled in mitochondrial depletion syndromes, such as those caused by a mutation in thymidine kinase 2 (TK2). Garone et al. investigated the effect of dCMP and dTMP supplementation in a TK2 mouse model. This supplementation bypassed the TK2 defect, increasing dTTP levels, and ameliorated biochemical abnormalities in these mice [68].

#### Reproductive Options

In addition to a wide variety of treatment options, reproductive options are also available. Patients who have been diagnosed with a pathogenic mutation in either nDNA or mtDNA known to cause mitochondrial disease have a number of reproductive options that aim to reduce the risk of transmitting the disease to their offspring. One option is oocyte or sperm donation (depending upon which parent is affected) which can prevent the inheritance of mitochondrial disease. An important consideration for some, however, is that the mother or father will not be genetically related to the offspring. Therefore, an alternative is prenatal diagnosis and preimplantation genetic diagnosis (PGD). These options have been used successfully to prevent transmission of nDNA and mtDNA mutations associated with mitochondrial disease [69, 70]. For the latter, the unique features of mtDNA mean that they will not be suitable for women who are homoplasmic for an mtDNA mutation or produce oocytes with high a mutation load.

The most recent reproductive option that may allow some women to have a genetically related child while reducing the risk of mitochondrial disease is mitochondrial donation (also known as mitochondrial replacement therapy). The novel IVF treatment involves transferring the mother’s nDNA from an affected egg into an enucleated egg from an unaffected donor, resulting in an embryo containing nDNA from both parents but mostly wild-type mtDNA from the healthy donor and a much lower risk of mitochondrial disease. The technique can be performed either before or after fertilization. Before fertilization, maternal spindle transfer (MST) or polar body transfer (PBT) can be used, while in fertilized eggs pronuclear transfer (PNT) can be used [71, 72]. Preclinical research studies have been important in confirming the potential of these techniques to reduce the risk of mitochondrial disease [73–75] and addressing the safety and efficacy of mitochondrial donation before clinical implementation [76].

Mitochondrial donation was approved for clinical use in the UK following an extensive policy process that led to the mitochondrial donation regulations being passed into law in March 2015 [77]. This legislative change allows the Human Fertilisation and Embryology Authority (HFEA) to issue licences to fertility centers who want to offer mitochondrial donation as a novel IVF treatment to reduce the risk of mitochondrial disease. To ensure strict regulation, license applications must be approved on a case-by-case basis for every patient and will only be considered when certain criteria are fulfilled as outlined by the HFEA. As this option is only applicable for mitochondrial diseases caused by mtDNA mutations, it could potentially benefit approximately 150 women each year in the UK [78].

### Concluding Remarks

Mitochondrial myopathies are progressive myopathies caused by the impairment of oxidative phosphorylation (OXPHOS). Alongside the traditional histochemical, immunohistochemical, and biochemical assays, the diagnosis of mitochondrial myopathies has been revolutionized by the introduction of NGS. NGS allows for a high-throughput screening of mtDNA and nDNA. However, there are currently no effective or disease-modifying treatments available for mitochondrial myopathies to halt the progression of the disease. Instead, existing therapeutic options have been focusing on the symptomatic management of disease manifestations. The development of large cohorts of patients with mitochondrial disease is enabling extensive studies to investigate the therapeutic effects of a variety of compounds shown to be of potential value in animal models. New molecular strategies, namely mtZFNs and mtTALENs, that cause beneficial heteroplasmic shifts in cell lines harboring varying pathogenic mtDNA mutations offer hope for the future. Moreover, recent developments in the reproductive options for patients with mitochondrial myopathies mean that for most families, the possibility of preventing transmission of the mutation to the next generation is now possible.

## Electronic supplementary material

ESM 1 (PDF 1224 kb)

## References

[1] Gorman GS, Chinnery PF, DiMauro S (2016). Mitochondrial diseases. Nat Rev Dis Primers.

[2] Shoffner JM, Lott MT, Voljavec AS, Soueidan SA, Costigan DA, Wallace DC (1989). Spontaneous Kearns-Sayre/chronic external ophthalmoplegia plus syndrome associated with a mitochondrial DNA deletion: a slip-replication model and metabolic therapy. Proc Natl Acad Sci U S A.

[3] Rossignol R, Faustin B, Rocher C, Malgat M, Mazat JP, Letellier T (2003). Mitochondrial threshold effects. Biochem J.

[4] Smith AC, Robinson AJ (2016). MitoMiner v3.1, an update on the mitochondrial proteomics database. Nucleic Acids Res.

[5] Sommerville EW, Chinnery PF, Gorman GS, Taylor RW (2014). Adult-onset Mendelian PEO Associated with Mitochondrial Disease. J Neuromuscul Dis.

[6] Gorman GS, Elson JL, Newman J (2015). Perceived fatigue is highly prevalent and debilitating in patients with mitochondrial disease. Neuromuscul Disord.

[7] Alston CL, Rocha MC, Lax NZ, Turnbull DM, Taylor RW (2017). The genetics and pathology of mitochondrial disease. J Pathol.

[8] Moraes CT, Ricci E, Bonilla E, DiMauro S, Schon EA (1992). The mitochondrial tRNA(Leu(UUR)) mutation in mitochondrial encephalomyopathy, lactic acidosis, and strokelike episodes (MELAS): genetic, biochemical, and morphological correlations in skeletal muscle. Am J Hum Genet.

[9] Bua E, Johnson J, Herbst A (2006). Mitochondrial DNA–Deletion Mutations Accumulate Intracellularly to Detrimental Levels in Aged Human Skeletal Muscle Fibers. Am J Hum Genet.

[10] Campbell G, Krishnan KJ, Deschauer M, Taylor RW, Turnbull DM (2014). Dissecting the mechanisms underlying the accumulation of mitochondrial DNA deletions in human skeletal muscle. Hum Mol Genet.

[11] Rygiel Karolina A, Picard M, Turnbull Doug M (2016). The ageing neuromuscular system and sarcopenia: a mitochondrial perspective. J Physiol.

[12] Ahmed ST, Alston CL, Hopton S (2017). Using a quantitative quadruple immunofluorescent assay to diagnose isolated mitochondrial Complex I deficiency. Sci Rep.

[13] Rocha MC, Grady JP, Grünewald A (2015). A novel immunofluorescent assay to investigate oxidative phosphorylation deficiency in mitochondrial myopathy: understanding mechanisms and improving diagnosis. Sci Rep.

[14] Bernier FP, Boneh A, Dennett X, Chow CW, Cleary MA, Thorburn DR (2002). Diagnostic criteria for respiratory chain disorders in adults and children. Neurology.

[15] Taylor RW, Pyle A, Griffin H (2014). Use of whole-exome sequencing to determine the genetic basis of multiple mitochondrial respiratory chain complex deficiencies. JAMA.

[16] Frazier AE, Thorburn DR, Compton AG. Mitochondrial energy generation disorders: genes, mechanisms and clues to pathology. J Biol Chem, (2017). 10.1074/jbc.R117.809194 PMC6462508 29233888

[17] Grady JP, Pickett SJ. mtDNA heteroplasmy level and copy number indicate disease burden in m.3243A>G mitochondrial disease. 10(6) (2018). 10.15252/emmm.201708262 PMC5991564 29735722

[18] Calvo SE, Compton AG, Hershman SG (2012). Molecular Diagnosis of Infantile Mitochondrial Disease with Targeted Next-Generation Sequencing. Sci Transl Med.

[19] Calvo S, Jain M, Xie X (2006). Systematic identification of human mitochondrial disease genes through integrative genomics. Nat Genet.

[20] McFarland R, Taylor RW, Turnbull DM (2010). A neurological perspective on mitochondrial disease. Lancet Neurol.

[21] Grady JP, Campbell G, Ratnaike T (2014). Disease progression in patients with single, large-scale mitochondrial DNA deletions. Brain.

[22] Volpi L, Ricci G, Orsucci D (2011). Metabolic myopathies: functional evaluation by different exercise testing approaches. Musculoskelet Surg.

[23] Tarnopolsky MA, Baker SK, Myint T, Maxner CE, Robitaille J, Robinson BH (2004). Clinical variability in maternally inherited leber hereditary optic neuropathy with the G14459A mutation. Am J Med Genet A.

[24] Suomalainen A, Elo JM, Pietilainen KH (2011). FGF-21 as a biomarker for muscle-manifesting mitochondrial respiratory chain deficiencies: a diagnostic study. *The Lancet*. Neurology.

[25] Lehtonen JM, Forsstrom S, Bottani E (2016). FGF21 is a biomarker for mitochondrial translation and mtDNA maintenance disorders. Neurology.

[26] Morovat A, Weerasinghe G, Nesbitt V (2017). Use of FGF-21 as a Biomarker of Mitochondrial Disease in Clinical Practice. J Clin Med.

[27] Yatsuga S, Fujita Y, Ishii A (2015). Growth differentiation factor 15 as a useful biomarker for mitochondrial disorders. Ann Neurol.

[28] Fujita Y, Ito M, Kojima T, Yatsuga S, Koga Y, Tanaka M (2015). GDF15 is a novel biomarker to evaluate efficacy of pyruvate therapy for mitochondrial diseases. Mitochondrion.

[29] Taivassalo T, Abbott A, Wyrick P, Haller Ronald G (2002). Venous oxygen levels during aerobic forearm exercise: An index of impaired oxidative metabolism in mitochondrial myopathy. Ann Neurol.

[30] Horvath R, Czermin B, Gulati S (2012). Adult-onset cerebellar ataxia due to mutations in CABC1/ADCK3. J Neurol Neurosurg Psychiatry.

[31] Glover Elisa I, Martin J, Maher A, Thornhill Rebecca E, Moran Gerald R, Tarnopolsky Mark A (2010). A randomized trial of coenzyme Q10 in mitochondrial disorders. Muscle Nerve.

[32] Stacpoole PW, deGrauw TJ, Feigenbaum AS (2012). Design and Implementation of the First Randomized Controlled Trial of Coenzyme Q(10) in Children with Primary Mitochondrial Diseases. Mitochondrion.

[33] Giorgio V, Petronilli V, Ghelli A (2012). The effects of idebenone on mitochondrial bioenergetics. Biochim Biophys Acta.

[34] Klopstock T, Yu-Wai-Man P, Dimitriadis K (2011). A randomized placebo-controlled trial of idebenone in Leber's hereditary optic neuropathy. Brain.

[35] Kaufmann P, Hirano M. Study of idebenone in the treatment of mitochondrial encephalopathy lactic acidosis & stroke-like episodes (MELAS); ClinicalTrials.gov, NCT00887562. 2009. (2009).

[36] Hirano M. Study of idebenone in the treatment of mitochondrial encephalopathy lactic acidosis & stroke-like episodes (MELAS); clinicaltrials.gov, NCT00887562. 2012. (2012).

[37] Cantó C, Houtkooper RH, Pirinen E (2012). The NAD(+) precursor nicotinamide riboside enhances oxidative metabolism and protects against high-fat diet induced obesity. Cell Metab.

[38] Bieganowski P, Brenner C (2004). Discoveries of nicotinamide riboside as a nutrient and conserved NRK genes establish a Preiss-Handler independent route to NAD+ in fungi and humans. Cell.

[39] Khan NA, Auranen M, Paetau I (2014). Effective treatment of mitochondrial myopathy by nicotinamide riboside, a vitamin B3. EMBO Mol Med.

[40] Tyynismaa H, Mjosund KP, Wanrooij S (2005). Mutant mitochondrial helicase Twinkle causes multiple mtDNA deletions and a late-onset mitochondrial disease in mice. Proc Natl Acad Sci U S A.

[41] van de Weijer T, Phielix E, Bilet L (2015). Evidence for a direct effect of the NAD+ precursor acipimox on muscle mitochondrial function in humans. Diabetes.

[42] Yatsuga S, Suomalainen A (2012). Effect of bezafibrate treatment on late-onset mitochondrial myopathy in mice. Hum Mol Genet.

[43] Viscomi C, Bottani E, Civiletto G (2011). In vivo correction of COX deficiency by activation of the AMPK/PGC-1alpha axis. Cell Metab.

[44] Reisman SA, Lee CY, Meyer CJ, Proksch JW, Sonis ST, Ward KW (2014). Topical application of the synthetic triterpenoid RTA 408 protects mice from radiation-induced dermatitis. Radiat Res.

[45] Shen W, Liu K, Tian C (2008). R-alpha-lipoic acid and acetyl-L-carnitine complementarily promote mitochondrial biogenesis in murine 3T3-L1 adipocytes. Diabetologia.

[46] Ahola-Erkkila S, Carroll CJ, Peltola-Mjosund K (2010). Ketogenic diet slows down mitochondrial myopathy progression in mice. Hum Mol Genet.

[47] Santra S, Gilkerson RW, Davidson M, Schon EA (2004). Ketogenic treatment reduces deleted mitochondrial DNAs in cultured human cells. Ann Neurol.

[48] Ahola S, Auranen M, Isohanni P (2016). Modified Atkins diet induces subacute selective ragged-red-fiber lysis in mitochondrial myopathy patients. EMBO Mol Med.

[49] Alam NM, Mills WC, Wong AA, Douglas RM, Szeto HH, Prusky GT (2015). A mitochondrial therapeutic reverses visual decline in mouse models of diabetes. Dis Model Mech.

[50] Szeto HH, Birk AV (2014). Serendipity and the Discovery of Novel Compounds That Restore Mitochondrial Plasticity. Clin Pharmacol Ther.

[51] Siegel MP, Kruse SE, Percival JM (2013). Mitochondrial-targeted peptide rapidly improves mitochondrial energetics and skeletal muscle performance in aged mice. Aging Cell.

[52] Karaa A, Haas R, Goldstein A, Vockley J, Weaver WD, Cohen BH (2018). Randomized dose-escalation trial of elamipretide in adults with primary mitochondrial myopathy. Neurology.

[53] Koene S, Spaans E, Van Bortel L (2017). KH176 under development for rare mitochondrial disease: a first in man randomized controlled clinical trial in healthy male volunteers. Orphanet J Rare Dis.

[54] Taivassalo T, Shoubridge EA, Chen J (2001). Aerobic conditioning in patients with mitochondrial myopathies: physiological, biochemical, and genetic effects. Ann Neurol.

[55] Jeppesen TD, Schwartz M, Olsen DB (2006). Aerobic training is safe and improves exercise capacity in patients with mitochondrial myopathy. Brain.

[56] Taivassalo T, Gardner JL, Taylor RW (2006). Endurance training and detraining in mitochondrial myopathies due to single large-scale mtDNA deletions. Brain.

[57] Taivassalo T, Jensen TD, Kennaway N, DiMauro S, Vissing J, Haller RG (2003). The spectrum of exercise tolerance in mitochondrial myopathies: a study of 40 patients. Brain.

[58] Cejudo P, Bautista J, Montemayor T (2005). Exercise training in mitochondrial myopathy: a randomized controlled trial. Muscle Nerve.

[59] Murphy JL, Blakely EL, Schaefer AM (2008). Resistance training in patients with single, large-scale deletions of mitochondrial DNA. Brain.

[60] Bates MGD, Newman JH, Jakovljevic DG (2013). Defining cardiac adaptations and safety of endurance training in patients with m.3243A>G-related mitochondrial disease()()(). Int J Cardiol.

[61] Zeviani M (2008). Train, train, train! No pain, just gain. Brain.

[62] Minczuk M, Papworth MA, Miller JC, Murphy MP, Klug A (2008). Development of a single-chain, quasi-dimeric zinc-finger nuclease for the selective degradation of mutated human mitochondrial DNA. Nucleic Acids Res.

[63] Bacman SR, Williams SL, Pinto M, Moraes CT (2014). The Use of Mitochondria-Targeted Endonucleases to Manipulate mtDNA. Methods Enzymol.

[64] Gammage PA, Rorbach J, Vincent AI, Rebar EJ, Minczuk M (2014). Mitochondrially targeted ZFNs for selective degradation of pathogenic mitochondrial genomes bearing large-scale deletions or point mutations. EMBO Mol Med.

[65] Hashimoto M, Bacman SR, Peralta S (2015). MitoTALEN: A General Approach to Reduce Mutant mtDNA Loads and Restore Oxidative Phosphorylation Function in Mitochondrial Diseases. Mol Ther.

[66] Gammage PA, Gaude E, Van Haute L (2016). Near-complete elimination of mutant mtDNA by iterative or dynamic dose-controlled treatment with mtZFNs. Nucleic Acids Res.

[67] Reddy P, Ocampo A, Suzuki K (2015). Selective elimination of mitochondrial mutations in the germline by genome editing. Cell.

[68] Garone C, Garcia-Diaz B, Emmanuele V (2014). Deoxypyrimidine monophosphate bypass therapy for thymidine kinase 2 deficiency. EMBO Mol Med.

[69] Nesbitt V, Alston CL, Blakely EL (2014). A national perspective on prenatal testing for mitochondrial disease. Eur J Hum Genet.

[70] Smeets HJ, Sallevelt SC, Dreesen JC, de Die-Smulders CE, de Coo IF (2015). Preventing the transmission of mitochondrial DNA disorders using prenatal or preimplantation genetic diagnosis. Ann N Y Acad Sci.

[71] Craven L, Tuppen HA, Greggains GD (2010). Pronuclear transfer in human embryos to prevent transmission of mitochondrial DNA disease. Nature.

[72] Lyndsey C, Charlotte LA, Robert WT, Doug MT (2017). Recent Advances in Mitochondrial Disease. Annu Rev Genomics Hum Genet.

[73] Yamada M, Emmanuele V, Sanchez-Quintero Maria J (2016). Genetic Drift Can Compromise Mitochondrial Replacement by Nuclear Transfer in Human Oocytes. Cell Stem Cell.

[74] Kang E, Wu J, Gutierrez NM (2016). Mitochondrial replacement in human oocytes carrying pathogenic mitochondrial DNA mutations. Nature.

[75] Hyslop LA, Blakeley P, Craven L (2016). Towards clinical application of pronuclear transfer to prevent mitochondrial DNA disease. Nature.

[76] Greenfield A, Braude P, Flinter F, Lovell-Badge R, Ogilvie C, Perry ACF (2017). Assisted reproductive technologies to prevent human mitochondrial disease transmission. Nat Biotechnol.

[77] Craven L, Herbert M, Murdoch A, Murphy J, Lawford Davies J, Turnbull DM (2016). Research into Policy: A Brief History of Mitochondrial Donation. Stem Cells.

[78] Gorman GS, Grady JP, Turnbull DM (2015). Mitochondrial donation--how many women could benefit?. N Engl J Med.

